# The 9th Santorini Conference: Systems Medicine, Personalised Health and Therapy. “The Odyssey from Hope to Practice”, Santorini, Greece, 30 September–3 October 2018

**DOI:** 10.3390/jpm8040043

**Published:** 2018-12-12

**Authors:** Sophie Visvikis-Siest, Vesna Gorenjak, Maria G. Stathopoulou, Alexandros M. Petrelis, Georges Weryha, Christine Masson, Brigitte Hiegel, Satish Kumar, Robert Barouki, Eric Boerwinkle, Georges Dagher, Panagiotis Deloukas, Federico Innocenti, John Lamont, Michael Marschler, Heiko Meyer, Urs A. Meyer, Charity Nofziger, Markus Paulmichl, Cora Vacher, Lynn Webster

**Affiliations:** 1 Université de Lorraine, Inserm, IGE-PCV, 54000 Nancy, France; vesna.gorenjak@inserm.fr (V.G.); maria.stathopoulou@inserm.fr (M.G.S.); apetrelis@live.com (A.M.P.); g.weryha@chru-nancy.fr (G.W.); christine.masson@univ-lorraine.fr (C.M.); brigitte.hiegel@univ-lorraine.fr (B.H.); satish.kumar@univ-lorraine.fr (S.K.); 2 INSERM UMR-S 1124, Université Paris Descartes, Université Sorbonne Paris Cité, 75270 Paris, France; robert.barouki@parisdescartes.fr; 3 Service de Biochimie Métabolomique et Protéomique, Hôpital Universitaire Necker Enfants Malades, AP-HP, 75015 Paris, France; 4 Department of Epidemiology, Human Genetics and Environmental Sciences, UTHealth School of Public Health, Houston, TX 77030, USA; Eric.Boerwinkle@uth.tmc.edu; 5 Inserm UMR-S 1124, Paris-Descartes University, 75270 Paris, France; georges.dagher@inserm.fr; 6 Department of Clinical Pharmacology, Queen Mary University of London, London E1 4NS, UK; p.deloukas@qmul.ac.uk; 7 Pharmacogenomics and Individualized Therapy, University of North Carolina Chapel Hill, Chapel Hill, NC 27599-2200, USA; innocent@email.unc.edu; 8 Randox Laboratories Ltd., Crumlin, County Antrim BT29 4XL, UK; john.lamont@randox.com; 9 PRAHealthSciences, 68165 Mannheim, Germany; marschlermichael@prahs.com; 10 Agena Bioscience GmbH, 22761 Hamburg, Germany; heiko.meyer@agenabio.com; 11 Biozentrum, University of Basel, 4056 Basel, Switzerland; urs-a.meyer@unibas.ch; 12 PharmGenetix Gmbh, 1090 Vienna, Austria; charity.nofziger@pharmgenetix.com; 13 Center for Health & Bioresources, Austrian Institute of Technology, 1210 Vienna, Austria; paulmichl@me.com; 14 NESMOS Department, University of Rome Sapienza, 00185 Rome, Italy; 15 Illumina, 81669 Munich, Germany; cvacher@illumina.com; 16 PRAHealthSciences, Salt Lake City, UT 84124, USA; WebsterLynn@prahs.com

**Keywords:** systems medicine, personalised medicine, pharmacogenomics, clinical trials, cardio-metabolic diseases, cancer, genetic screening, “-OMICs” biomarkers, santorini conference

## Abstract

The 9th traditional biannual conference on Systems Medicine, Personalised Health & Therapy—“The Odyssey from Hope to Practice”, inspired by the Greek mythology, was a call to search for practical solutions in cardio-metabolic diseases and cancer, to resolve and overcome the obstacles in modern medicine by creating more interactions among disciplines, as well as between academic and industrial research, directed towards an effective ‘roadmap’ for personalised health and therapy. The 9th Santorini Conference, under the Presidency of Sofia Siest, the director of the INSERM U1122; IGE-PCV (www.u1122.inserm.fr), University of Lorraine, France, offered a rich and innovative scientific program. It gathered 34 worldwide distinguished speakers, who shared their passion for personalised medicine with 160 attendees in nine specific sessions on the following topics: First day: The Odyssey from hope to practice: Personalised medicine—landmarks and challenges Second day: Diseases to therapeutics—genotype to phenotype an “-OMICS” approach: focus on personalised therapy and precision medicine Third day: Gene-environment interactions and pharmacovigilance: a pharmacogenetics approach for deciphering disease “bench to clinic to reality” Fourth day: Pharmacogenomics to drug discovery: a big data approach and focus on clinical data and clinical practice. In this article we present the topics shared among the participants of the conference and we highlight the key messages.

## 1. The 9th Santorini Conference

The 9th traditional biannual conference on Systems Medicine, Personalised Health and Therapy, under the auspices of the International Federation of Clinical Chemistry and Laboratory Medicine (IFCC), the European Federation of Clinical Chemistry and Laboratory Medicine (EFLM), the Hellenic Society for Basic and Clinical Pharmacology (EEI), the Hellenic Society of Pharmacogenomics and personalised Diagnosis and Therapy (EEPHARM), and the European Society of Pharmacogenomics and Personalised Therapy (ESPT), took place in Santorini, Greece, on 30 September 2018.

It was organised with the help of different industrial companies: Randox (Crumlin, UK) (loyal Gold sponsor)—Siemens Healthineers (Erlangen, Germany), Illumina (Munich, Germany), Agena Bioscience (Hamburg, Germany), DiaSys (Connecticut, États-Unis) (Silver sponsors)—the Banque Populaire (Paris, France), Mastiha Growers Association (Chios, Greece), PharmGenetix (Vienna, Austria), and HMG systems Engineering (Fuerth, Germany) (Other sponsors).

**Figure 1:**
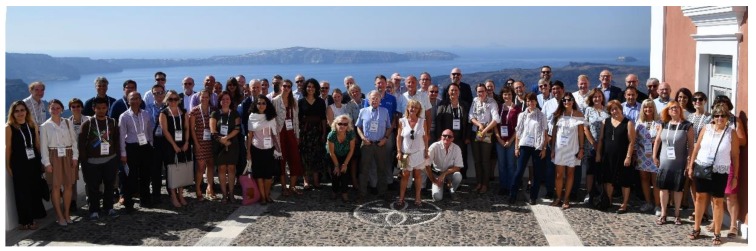
Participants of The 9th Santorini conference in Petros M. Nomikos Conference Centre. (Photo by George Mindrinos).

The 9th Santorini Conference, under the Presidency of Sofia Siest, the director of the INSERM U1122; IGE-PCV (www.u1122.inserm.fr), University of Lorraine, France, offered a rich and innovative scientific program. It gathered 34 worldwide distinguished speakers, who shared their passion for personalised medicine with 160 attendees (Figure 1) in nine specific sessions on the following topics:

- First day: The Odyssey from hope to practice: Personalised medicine—landmarks and challenges
- Second day: Diseases to therapeutics—genotype to phenotype an “-OMICS” approach: Focus on personalised therapy and precision medicine
- Third day: Gene–environment interactions and pharmacovigilance: A pharmacogenetics approach for deciphering disease “bench to clinic to reality”
- Fourth day: Pharmacogenomics to drug discovery: A big data approach and focus on clinical data and clinical practice.

In this article, we present the topics shared among the participants of the conference and we highlight the key messages.

### 1.1. The Odyssey from Hope to Practice

The conference was officially open with the welcome by Sofia Siest, who made a brief summary on the evolution of this colloquium over the past 16 years, initiated in 2002, thus being the oldest International conference in the field of personalised Medicine medicine and pharmacogenomics and one of the most important conferences on genetic predisposition to health, diseases, and response to drugs and environment. After 16 years, the 9th Santorini Conference “Systems Medicine and Personalised Health and Therapy” aimed to reflect on the frontiers of current genomic knowledge in the fields of cardiometabolic diseases and cancer and to reveal the practical use of this knowledge in disease prevention, diagnosis, and pharmacogenomics aiming to directly impact the socioeconomic aspects of public health. Sophia Siest’s presentation was inspired by the Odyssey, the Greek ancient epic poem of Homer, and she embarked with the attendees on a ship from Troy (Hope) to Ithaca (Practice), coursing through the history of the momentous events and achievements that paved the way for personalised medicine. She set sail amidst important genetic discoveries and obstacles that are slowing the full implementation of accumulated knowledge into everyday practice, beginning with the discovery of the first human genome and voyaging through the projects that contributed to the progress of personalised medicine. Her introduction was illustrated with an animated movie (available on https://www.youtube.com/watch?v=SReS8WmH2jU) presenting the journey of Odysseus on his way to Ithaca—from hope to practice—whilst reminding of great achievements that contributed enormously to the development of personalised medicine. The whole story describing the history of personalised medicine was carefully gathered in the eponymous article [1].

Following the opening words of Sofia Siest, the keynote lecture, presented by Eric Boerwinkle (Houston, TX, USA) carried on with the journey to personalised medicine and unravelled the landmarks and challenges of the field. He discussed the necessity of genome-wide association studies (GWAS) and their input in the discovery of new genes, but the inclusion of ethic variations is also necessary for taking knowledge further. He pointed out that apart from genes, their interactions with environment through time should be actively considered in genomic research. He also discussed pharmacogenomics and the importance of including genomic information in medical guidelines. The importance of technological advances was also mentioned as a lesson learned even from Homer’s era. He concluded that Ithaca for personalised medicine should be based on relating clinicians and researchers and include improved sequencing to improve diagnostics and personalisation of therapy of severe and chronic diseases.

## 2. Conference Sessions

### 2.1. “-OMICs” Biomarkers Commonality in Cardiometabolic Diseases and Cancer—Can Sequencing Offer a Diagnosis?

#### Chairs: Panagiotis Deloukas, London, UK/Heiko Meyer, Hamburg, Germany

The session started with the morning lecture, given by Christopher B. Newgard (Durham, NC, USA), who discussed the application of metabolomics and other “-omics” tools for the understanding of mechanisms contributing to pandemic metabolic diseases of our era—diabetes, obesity, and cardiovascular disease [2]. With the practical examples from the investigation of the mechanistic and therapeutic significance of a metabolomics signature of perturbed branched chain amino acid (BCAA) catabolism in multiple cohorts of insulin resistant humans, compared to normally insulin sensitive controls [3], Dr Newgard demonstrated the potential of metabolic profiling for defining novel metabolic disease mechanisms and new therapeutic strategies.

Next, two remarkable projects that evolved the era of genomics were presented. Firstly, Mark Caulfield (London, UK) spoke about the 100,000 Genomes Project and its impact on transforming genomics in healthcare. In the frames of the project, thirteen National Health Service (NHS) Genomic Medicine Centres of excellence across England were created in collaboration with the NHS to enable the generation of clinical data and sample flows. Moreover, in partnership with Illumina, one of the largest next generation sequencing centres in the world was created to generate the highest fidelity and most comprehensive whole genome DNA sequence produced from patients to date with high fidelity clinical data stored in a de-identified format within a multipetabyte data infrastructure.

Secondly, Peter Campbell (Hinxton, UK) from The Welcome Trust Sanger Institute introduced The International Cancer Genome project. The main aim is to provide important insights into the biology of cancer through discovering the genes that are frequently mutated in tumours, and with studying the patterns of mutations seen in cancer cells. Overall, the projects explore basic scientific questions on the role that somatic mutations play in clonal evolution, ageing, and development.

The first session of the conference was completed with the lecture of Eleftherios Diamandis (Toronto, ON, Canada) on personalised biomarkers in cancer. A slow progress in cancer biomarker discovery and general speculations of scientists, who believed it is unlikely to discover new serological biomarkers characterised by high sensitivity and specificity, encouraged researchers to propose a new way of improving the landscape of cancer biomarker research. Screening new patients for a large number of previously described biomarkers with high specificity (>90%) but low sensitivity (<30%) could identify new informative markers for clinical use, important for managing individual cancer patients. Moreover, this approach may explain the reasons for the increased value of some biomarkers that appear only in a small group of patients. These differences in expression are likely to be linked to specific genomic alterations, which could then be found with genomic sequencing [4].

### 2.2. Unmet Clinical Needs in the Prevention and Treatment of Cardiometabolic Diseases and Cancer-Comorbidities

#### Chairs: Georges Weryha, Nancy, France/John Lamont, Crumlin, County Antrim, UK

For the introduction to the new session, Manuel Rosa Garrido (Los Angeles, CA, USA) shared his findings in the epigenetics of cardiovascular diseases and comorbidities. Epigenetic mechanisms control gene expression at the individual locus scale as well as at the genomic scale via the formation of long-range regulatory interactions and the formation of chromosome territories. The presented case-control study on mice aimed to determine the impact of such mechanisms during the development of heart failure. Manuel Rosa Garrido proposed that global remodelling of chromatin after traverse aortic constriction and 11-zinc finger protein (CTCF) depletion drives heart failure. He concluded that heart failure involves conserved structural reprogramming of chromatin microenvironments with histone marks and DNA methylation, which play an important role in controlling expression.

In his speech, Abraham Aviv (New Jersey, USA) focused on the role of telomere length (TL) in the etymology of chronic diseases. He pointed out that short TLs have a protective role against cancer, while the trade-off for it might be susceptibility to cardiovascular diseases CVD, suggesting TL as a common factor of these diseases. Furthermore, associations manifest at the genomic level, as single nucleotide polymorphisms (SNPs) associated with short leucocyte telomere length (LTL) are also associated with CVD and SNPs associated with long leucocyte TL are also associated with several cancers. Therefore, he concluded that TL might play a causal role in CVD and some cancers. Finally, these findings also have considerable ramifications for the longevity of humans.

### 2.3. Pipeline Challenges in Precision Medicine—From Patient Sampling to a Clinical Report

#### Chairs: Georges Dagher, Paris, France/Charity Nofziger, Salzburg, Austria

Markus Paulmichl (Salzburg, Austria) presented the Austrian experience of a good practice in carrying patient data in the clinical report. He presented the national program of the Austrian government that aims for the increased implementation of pharmacogenomics into the daily clinical routine. He discussed the different steps for reporting pharmacogenomics data to clinicians, which can be challenging, especially in cases of polytherapy. A schematic report warns clinicians about inappropriate medications and proposes possible alternative solutions.

The importance of standardised pre-analytical workflows that can ensure good quality samples for reliable analytical test results was recognised by EU. The result was the SPIDIA consortium that gathered academic institutions, international organisations, and private companies, in order to identify variables in the pre-analytical phase that can influence the quality of samples and should be controlled to guarantee a robust diagnosis. Uwe Oelmueller (Hilden, Germany) gave the overview on the new EU SPIDIA4P project and its potential for developing on these basic pre-analytical workflow standards that guide the laboratories workflow parameters and guarantees analytical test results. In relation to these findings, 22 standards are being drafted in the scope of SPIDIA4P. Ten of them are already published by the European Committee for Standardization as European CEN documents and now being further broadened to be published as International standards (ISO).

In relation to the previous speaker, Karl Friedrich Becker (Munich, Germany) gave the example of implementation of standardised pre-analytical CEN/TS and ISO/IS documents based on SPIDIA and SPIDIA4P projects in the current workflows, in order to provide a successful integration of proteomic studies in the clinic. He discussed the influences of sample processing, including sample collection, transport, stabilisation, and storage and analyte (e.g., protein) extraction on the final assay result, and highlighted the importance of standardisation of the entire workflow from test ordering to the report of the molecular assay, with special emphasis on the pre-analytical phase.

In addition to pre-analytical challenges in sample procession, there are other obstacles that can hinder the usage of patient samples for precision medicine. Circulating tumour cells firstly showed a potential utility as a surrogate biomarker of tumour biology via a liquid biopsy. Maria G. Daidone (Milan, Italy) presented the challenges in the use of such liquid biopsies for personalised medicine.

### 2.4. PGx-Regulatory Perspectives and Future Technical/Analytical Developments

#### Chairs: Markus Paulmichl, Salzburg, Austria/Lynn Webster, Salt Lake City, UT, USA

In personalised medicine, genomic data became an important source of information for the evaluation of efficacy and safety of medicinal products. Similar to standardisation of pre-analytical workflows to ensure the good quality of samples for reliable analysis, the use of genomic biomarkers in drug development should, in order to be of value, follow certain principles. Markus Paulmichl (Salzburg, Austria) presented the Good Pharmacogenomic Practice guideline from the pharmacogenomics working group within the European Medicines Agency (EMA), which provides recommendations for the realisation of genomic studies in relation to medical therapy.

To conclude the last daily session, Simone Vanoni (Salzburg, Austria), discussed how correct and reliable determination of *CYPD26* functional activity is extremely complex and needs careful evaluation. The in vitro assay developed at PharmGenetix, combined with extensive genotyping, will allow for a fast and precise definition of the activity of various *CYP2D6* alleles.

### 2.5. Gene-Environment Interactions in Cardio-Metabolic Diseases and Cancer

#### Chairs: Robert Barouki, Paris, France/Michael Marschler, Mannheim, Germany

Georges Dedoussis (Athens, Greece) inaugurated the third day of the conference with a presentation on nutrigenetics in non-alcoholic fatty liver disease (NAFLD). This disease is primarily activated by dietary factors, obesity, and insulin resistance. However, a polygenic background is believed to impact susceptibility to the onset and progression of NAFLD. Therefore, the study of gene–diet interactions that lead to NAFLD onset and progression became an important subject of research and key to enabling the application of personalised nutrition and thus providing patients with more efficient therapies. Georges illustrated his presentation with very interesting examples of diet experience: Diet with increased fish intake, which would normally be considered as healthy, resulted in increased intrahepatic accumulation of triglycerides for the carriers of a TM6SF2 variant, one of the four genetic variants that have been identified as risk factors for NAFLD. Such findings demonstrate the importance of nutrigenetics research and application of the right diet for the patients with NAFLD.

Hugues Aschard (Paris, France) explained the theoretical aspects of interaction tests in regression models with the use of real data examples, including breast cancer, to illustrate the potential impact of having the right understanding of statistical analysis on detection and clinical utility of data. Hugues showed that the simplest biological interaction models—in which the magnitude of a genetic effect depends on a common exposure—are among the most difficult to identify. Moreover, he presented new insights on advantages and limitations of multivariate interaction models that can be leveraged for future method development and for the improvement of our understanding of the interplay between genetic variants and environmental exposures in multifactorial traits and diseases. The identification of precise and predictive biomarkers of health and disease is a critical objective of clinical biochemistry and biomedical research.

Robert Barouki (Paris, France) presented how recent developments in exposome studies and epigenomics could be used to support preventive action in medicine. Research in the exposome field allowed the development of sensors and biological biomarkers using “-omics” technologies that can support the prediction of the effect of those exposures on human health. Robert pointed out that precision medicine has primarily focused on adapting treatments to the genetic profiles of tumours, but in fact, it originally had a wider scope, including the use of robust biomarkers for disease prevention. Using different sensors, metabolomics and epigenomics, it now seems possible to generate precise observations that could be of value for prevention.

### 2.6. Latest Insights in Stroke: Clinical Trials and Applicability of ‘-OMICS’ Data in Patients’ Stratification

#### Chairs: John Lamont, Crumlin, County Antrim, UK/Federico Innocenti, Chapel Hill, NC, USA

As in many diseases, response to treatment in stroke differs from one individual to another. Pharmacogenetics aims to stratify patients based on their genetic information into groups that are more likely to benefit from a particular intervention, in order to select appropriate treatments. An increased understanding of stroke pharmacogenetics has been driven by advances in genotyping technology and increased interest in developing targeted pharmaceutical treatments. Guillaume Pare (Ontario, Canada) summarised the pharmacogenetics of stroke by providing an overview of the genetic variants that contribute to the individual responses to aspirin, clopidogrel, warfarin, and dabigatran and discussed considerations for evidence-based implementation. He suggested that the reasons for the limitations in implementation of pharmacogenetics in clinical settings can be found in the lack of awareness and need for evidence-based recommendations.

Alexander Haliassos (Athens, Greece) continued the discussion on stroke, mainly focusing on protein biomarkers in the diagnosis of ischemic stroke [5]. Fast and accurate diagnosis of patients is crucial but challenging, since ischemic stroke cannot be identified based only on clinical assessment. New non-invasive tests that could quickly distinguish stroke from stroke mimics and ischemic from haemorrhagic stroke would be a good alternative to CT or MRI imaging. Alexander Haliassos suggested that the identification of blood biomarkers of stroke is a prospective area of research, since their potential use is not limited to diagnosis and differentiation but can be applied to prognosis and patient treatment monitoring. However, he pointed out factors that are limiting such endeavours: The heterogeneity and complexity of stroke aetiology, analytical issues, and difficulties at the interpretation of laboratory measurements. To date, many biomarkers have been identified, but none so far have shown sufficient sensitivity and specificity to be used in the clinical setting.

### 2.7. Pain Management—A ‘Journey’ from ‘Clinical Trial’ via ‘Post-Marketing Pharmacovigilance/Risk Management’ to ‘Success Story’

#### Chairs: Michael Marschler, Mannheim, Germany/Lynn Webster, Salt Lake City, UT, USA

The session started with the journey towards successful pain management using genetic biomarkers as common denominators for specific pain disorders. Lynn Webster (Salt Lake City, UT, USA) stated that every clinician knows patients may develop very different degrees of pain and suffering after the same standard procedures. This interindividual variability is the rule rather than the exception.

The clinical problem is that this huge variability is unpredictable. There are multiple reasons for the variability, but an individual’s genotype is a major contributing factor. Genes have ethnicity-specific effects in response to pain. For example, the *GCH1* gene is protective for pain in a Caucasian population, and it is a risk factor for pain in African Americans with sickle cell pain. Then, there are genes that encode metabolic factors, and they have significant variation among ethnic populations. Thus, these ethnic-specific effects are common. Also very common are sex-specific effects of genes that are described for many pain genes. There are three scenarios of these sex-specific effects: The same genetic variations may have much stronger effects in one sex compared to another; they may have no effect in one sex compared to another; or they may have the opposite effect in females, for example, compared to males.

Genes also have combined effects on pain. For example, when analysing both the catechol-*O*-methyltransferase (*COMT*) and mu-opioid receptor (*OPRM1*) common-function SNPs, the morphine requirements can be much less than in patients without the common SNPs. In addition, the genetic biomarker, brain-derived neurotrophic factor gene (*BDNF*), has recently been shown to be associated with an increased risk of chronic postsurgical pain. The reason for this is unclear.

Dr Webster also described a large international study designed to see if genetic biomarkers for chronic low back pain (CLBP) could be identified. In a just published paper, the researchers reported that a meta-analysis of GWAS for CLBP identified and replicated genetic loci associated with CLBP (SOX5, CCDC26/GSDMC and DCC).

Laure Elens (Brussels, Belgium) continued the journey, presenting pharmacogenetics of opioid treatments. Interindividual differences in sensitivity to pain can be very problematic, especially during medical interventions and in managing discomfort after medical intervention. However, there is evidence that genetic variations and SNPs in genes, implicated in the opioid response pathway or the mechanisms of pain sensing, might explain the various phenotypes related to pain susceptibility and response to opioid therapy. Laure Elens summarised current knowledge in opioid pharmacogenetics and possible beneficial applications in clinical practice to allow better individualisation of pain discomfort when treated with opioids, and provided an overview of opioid pharmacogenetic studies and how it might be useful in understanding interindividual differences in pain perception and treatment effectiveness and/or drug-related toxicity, to determine whether genetic testing has any clinical significance in decision making for the management and control of pain, or not.

Noelia Martin Granado (Madrid, Spain) followed with a presentation about pharmacogenomics in post-marketing pharmacovigilance. Firstly, she reminded the audience that pharmacovigilance is the science and activities related to the detection, assessment, understanding, and prevention of adverse drug reactions (ADRs), since there is robust evidence that genomic factors may play a key role in the pathogenesis of both predictable and idiosyncratic ADRs. Over the past years, several studies have identified a number of common and rare variants that are associated with an increased risk of ADRs. In particular, polymorphisms in genes encoding drug transporters, drug-metabolising enzymes, and drug targets (e.g., enzymes, receptors) can lead to the occurrence of ADRs. As more reliable and affordable genetic testing tools become available, pharmacogenomics seems promising in terms of facilitating personalised drug therapy by maximising the therapeutic efficacy of drugs while minimising the occurrence of ADRs in patients. Due to the strong synergy existing between pharmacovigilance and pharmacogenomics disciplines, as both aim to understand the inter-relationships between drug therapeutic efficacy and safety, it has been proposed to put into practice pharmacogenovigilance, a combination of pharmacogenomics and pharmacovigilance: Pharmacovigilance activities fed and guided by accompanying pharmacogenomics analyses.

Andrew Purchase (Swansea, UK) finished the journey and discussed how the adoption of pharmacogenomics leads to patient benefit. He provided an overview of the increasing acceptance and adoption of pharmacogenomics into clinical practice, and how this translates into benefits for the patient. The presentation also explored methodologies for raising patient awareness and understanding of the potential benefits linked to pharmacogenomics, as well as current/future technologies that support the use of genomic data. Genomic data have emerged as a key component of precision medicine, offering a way for providers to determine the most effective therapies for patients based on the unique makeup of their DNA. As an example, Andrew informed the audience that before prescribing the antiviral drug Abacavir (Ziagen), doctors now routinely test HIV-infected patients for a genetic variant that makes them more likely to have a negative reaction to the drug. The need to test patients for the *HLA-B* (type 5701) gene and to remind patients to contact their doctor immediately if they develop symptoms is suggestive of hypersensitivity. Andrew concluded that with reduced costs, PGx has the potential to further change the landscape of healthcare and improve the treatment options for patients, whilst reducing/removing risk.

### 2.8. Pharmacogenomics: Challenges of Clinical Translation

#### Chairs: Urs A. Meyer, Basel, Switzerland/Charity Nofziger, Salzburg, Austria

The first session of the day started with a lecture of Federico Innocenti (Chapel Hill, NC, USA), who discussed the role of genomics in safety of drug treatment in oncology [6]. He reminded the audience about the importance that adverse events have on drug treatment in cancer. Not only do they have a significant burden on the quality of life of the patient and their families, but they also reduce confidence in the treatment and often lead to permanent discontinuation of therapy. However, genetic analyses can increase patient safety and improve the safety of cancer drugs in several ways. These aspects are of particular importance for implementation, as access to genetic profiling is becoming more common for patients.

Commonly, drugs frequently associated with adverse events are known to be metabolised by enzymes with genetic polymorphisms. Accurate genetic analysis of patients using such treatment is therefore of crucial importance to prevent possible adverse events. *CYP2D6* is responsible for metabolising around 25% of clinically prescribed drugs, and is riddled with function altering genetic variations, including point mutations, insertions and deletions, as well as hybrid formation with its pseudogene, *CYP2D7*—all of which make accurate genotyping quite challenging [7]. In the following speech, Charity Nofziger (Salzburg, Austria) presented the technical challenges in genotyping *CYP2D6*, focusing on allele drop-out events that can severely alter the prediction of metabolising phenotypes within a particular patient.

Urs A. Meyer (Basel, Switzerland) continued the debate with an overview of present studies on the clinical implementation of digital signatures of the drug response profiles. In most therapies, efficacy and toxicity is determined by the combined action of multiple genetic, epigenetic, environmental, and host factors, and the genetic contribution may be too small for predicting drug response. Therefore, application of “-omics”-based data in combination with clinical and environmental factors to predict an individual’s drug response is one of the visions of pharmacogenomics, personalised medicine, and precision medicine. For an increasing number of actionable gene–drug interactions, practice guidelines based on genetic and clinical information have been established (http://cpicpgx.org). The incorporation of pharmacogenetic test results, combined with clinical decision support (CDS) into machine-readable electronic medical records (EMRs) allows a clinical implementation of this information. In the view of the above, Urs discussed how to best apply the already existing digital genomic and other “-omics” information to optimise drug response.

Belgin Süsleyici (Istanbul, Turkey) presented an example of implementation of pharmacogenetics in Turkish clinical practice, focusing on the enzyme dihydropyrimidine dehydrogenase (DPD). DPD is an important catalyser of fluorouracil metabolism, a common drug for treatment of colorectal carcinoma. However, individuals carrying at least one copy of a loss of function DPYD variant may not be able to metabolise fluorouracil at normal rates and are at risk of life-threatening toxicity. In recent research, associations between fluorouracil treatment outcomes and germ line polymorphisms in DPD were analysed. Belgin demonstrated that the variances in the DPD gene can have no functional consequences on enzymatic activity, can decrease, or increase metabolism of the drug. She concluded that clinicians should be strongly encouraged to consider testing for DPD poor metaboliser variants as a rational pretreatment screening for patient candidates to a fluoropyrimidine-based therapy, in order to prevent toxicities.

For the closure of the session, Alexander Jetter (Zurich, Switzerland) shared his views on pharmacogenetics as the basis of individualised therapy decisions. He presented two clinical cases and discussed the choice of therapy for each presented case that was based on the genomic data of patients. For some medications, such as those containing carbamazepine, such a procedure is highly recommended. Moreover, different pharmacogenetic test approaches were presented. Alexander highlighted that pharmacogenetic tests should be used in combination with nongenetic information in order to treat “the right patient with the right drug in the right dose at the right time”.

### 2.9. What Can We Learn from Electronic Health Records?

#### Chairs: Panagiotis Deloukas, London, UK/ Markus Paulmichl, Salzburg, Austria

The final session was introduced by Harry Hemingway (London, UK) continued the session with a presentation of a large-scale enquiry of longitudinal electronic health records (EHR) linked to genomic and “-omics” resources [8]. Such resources can offer discovery science with well-established designs, improve care through preventive genomics, and provide new kinds of scientific enquiry based on an agnostic understanding of longitudinal human phenome sequences. He presented the national platform CALIBER (www.ucl.ac.uk/health-informatics-caliber) that developed phenotyping algorithms for 72 diseases and risk factors and made them available through an open-access data portal, together with a set of open source tools. He concluded that in the era of precision medicine, robust approaches to creating, validating, and sharing EHR-derived phenotypes are critical to enable cross-source analyses of thousands of simple and complex traits in millions of individuals. The CALIBER approach provides a transparent methodology for transforming raw EHRs to reproducible phenotypes for use in such studies.

Richard Trembath (London, UK) introduced the East London Genes and Health cohort, containing primary health data of over 20,000 people, aiming to improve health among people of Pakistani and Bangladeshi heritage in East London by analysing the genes and health of local people.

The last presentation of the conference was given by Panagiotis Deloukas (London, UK), who shared his experiences of linking genetic data to risk of recurrent cardiovascular events through hospital records. So far, 70 loci were associated with coronary heart disease and myocardial infraction and are currently undertaking further analyses in UK Biobank, including gene–gene and gene–environment interactions, together with an integrated approach of using CHD risk factors, such as lipid levels, blood pressure, haematological traits, and height, through QTL analyses in large, well-phenotyped cohorts with an ultimate goal of developing a risk model for recurrent MI and testing its clinical utility.

## 3. Oral Communications Session

### Chairs: Sofia Siest, Nancy, France/Eric Boerwinkle, Houston, USA

The conference invited young researchers and industrials to submit abstracts, to present their current work and win a reward, given by the organisation committee. Six participants were selected for 15 min oral presentations.

Said El Shamieh (Beirut, Lebanon): Genetic and protein profiling of cancer tumours, a first step towards personalised therapy.

Four cases of personalised therapy for cancer were presented. Molecular profiling has managed to provide better therapeutic options in 3 out of 4 of these cases. Furthermore, a guideline for a personalised treatment in endometrial cancer was presented.

Gilles Lunzenfichter (Luzern, Switzerland): Welcome to a new world of Intelligent Connected Care.

The presentation introduced us to M+ Intelligent Connected Care that enables synchronisation of patients’ data with physician devices and enables the live sharing of patients’ data and measurements. It is specialised in chronic conditions such as diabetes, hypertension, and cardiovascular and overweight indications.

Alexia Giannoula (Barcelona, Spain): Temporal comorbidity patterns in prostate cancer disease trajectories based on semantic, phenotypic, and genetic similarities.

Comorbodities and studies of molecular interactions were presented on prostate cancer patients. The aim was to unravel related pathogenetic processes and hidden diseases patterns.

Laurent Becquemont (Paris, France): Investigation of novel biomarkers of drug-induced kidney injury in renal transplant recipients undergoing graft biopsy.

New urinary biomarkers for drug-induced kidney injury were presented, in comparison to standard biomarkers. He concluded that new urinary biomarkers alone do not outperform the classical ones.

Bianca van den Bosch (Maastricht, The Netherlands): Evolution of Dihydropyrimidine Dehydrogenase (DPD) Diagnostics in a Single Center in a Time-Period of Eight Years.

The screening of the *DPYD* gene to detect variants related to change enzyme activity. She concluded that a combination of genetic screening and measurements of enzyme activity is better than these methods alone.

Carlos Malpica (Doha, Qatar): Bridging the Multi-Omics Precision Medicine Gap in the Middle East: The Valdia Health Experience.

Carlos discussed the implementation of precision medicine in Qatar and future challenges for establishing new networks of collaborations that will make this implementation possible.

The participants of the Santorini Conference voted for Bianca van den Bosch and Carlos Malpica to receive the Gerard SIEST Awards for the best oral presentations. Bianca van den Bosch obtained the award offered by the *Journal of Personalised Medicine* and Carlos Malpica the award offered by the University of Lorraine.

## 4. Posters

Sixty-three posters were presented that were classified in 2 groups (group 1: “-omics” biomarkers and group 2: Pharmacogenomics). Alzbeta Hlavackova received one “Gerard SIEST” award for the best poster in group 1 with the poster entitled “Changes in Plasma Metabolomic Profile during Acute Myeloid Leukemia Treatment” offered by De Gruyter publisher. Carolina Dagli-Hernandez won the second “Gerard SIEST” award for the best poster in group 2 with the poster entitled “Rare Variants in CYP2C9 and CYP3A5 Detected in Patients with Familial Hypercholesterolemia with Statin-Related Adverse Events” granted by the European Society of Pharmacogenomics and Personalised Therapy (ESPT).

## 5. Industrial Workshops

One of the important aims of the conference was also to establish strong relations among different sectors: Research–hospitals and industry. New improvements and techniques from innovative companies were presented during the conference.

### 5.1. Randox Workshop

Randox is a world leader in the in-vitro diagnostics industry, offering the most comprehensive insight into patient diagnosis, allowing for more effective disease management and treatment. The company developed biochip array technology (BAT), which is capable of simultaneous multianalyte diagnostic testing within the fields of clinical research and drugs of abuse testing. Cliona Johnston (Crumlin, County Antrim, UK) presented new possibilities for clinical evaluation of the type 1 diabetes based on genetic risk evaluation, using the innovative biochip array technology.

### 5.2. Illumina Workshop

Medical progress is vastly dependent upon tight collaboration between academy, hospitals, and industry. Illumina is an enterprise strongly involved in genetics and pharmacogenomics studies. The expert in pharmacogenomics Ron Van Schaik (Rotterdam, The Netherlands) discussed his experiences with pharmacogenetics using the Illumina GSA array. His speech was followed with a presentation by Lili Milani (Tartu, Estonia) on the Estonian personalised medicine initiative: A calculation of polygenic risk scores, pharmacogenetics, and rare mutations.

## 6. Closed Meetings

### 6.1. Closed Meeting—Mast4health Project

A closed meeting was reserved for the collaborators of the MAST4HEALTH project, a Marie Sklodowska-Curie Actions (MSCA) Research and Innovation Staff Exchange (RISE) program under the EU Horizon 2020 framework.

### 6.2. Closed Meeting—VEGF Consortium

The members of the VEGF Consortium (www.vegfconsortium.org) gathered together in a closed meeting. They hdiscussed the past work of the consortium and future projects, aiming to maintain and further develop the transnational collaborative network, dedicated to large integrative and multidisciplinary genomic studies of the Vascular Endothelial Growth Factor, in order to generate applicable knowledge for medical practice.

## 7. Conclusions and Perspective Remarks

The Conference officially finished with conclusions and perspective remarks given by Sofia Siest (Nancy, France) and Urs A. Meyer (Basel, Switzerland). They underlined the success of the conference, which, with pertinent illustrations and examples, successfully answered the questions that had been set:

⮚ Can genetic screening help to identify individuals at greatest risk for cardiometabolic diseases and cancer?
⮚ What is the greatest clinical need with regard to diagnosis, prediction, and patient stratification for these pathologies and how is this/can this be addressed?
⮚ Does a genetic risk score identify patients at the highest risk and is its use justified in clinical practice?
⮚ What are the challenges of the current clinical trials?
⮚ What about comorbidities with ageing?
⮚ What is the impact of pharmacogenomics on:
  - The deliverance of more predictable responses to drug therapy?
  - The minimisation of the occurrence and severity of adverse drug reactions?
  - The conduction of more cost-effective clinical trials?
  - Drug discovery and the drug development process?
Do the existing diagnostic tools answer the needs of pharmacogenomics?

Finally, Sofia Siest announced the next event—The 10th Santorini Conference, in 2020 (28 September–1 October).
